# In vitro combination of tigecycline with other antibiotics in Stenotrophomonas maltophilia isolates

**DOI:** 10.3906/sag-1808-55

**Published:** 2019-04-18

**Authors:** Dilek KARAMANLIOĞLU, Murat DİZBAY

**Affiliations:** 1 Clinic of Infectious Diseases and Clinical Microbiology, Dr. Nafiz Körez State Hospital, Sincan, Ankara Turkey; 2 Department of Infectious Diseases and Clinical Microbiology, School of Medicine, Gazi University, Beşevler, Ankara Turkey

**Keywords:** Combination, *Stenotrophomonas maltophilia*, synergy, tigecycline

## Abstract

**Background/aim:**

The aim of this study was to determine the usefulness of tigecycline in combination treatment of *Stenotrophomonas maltophilia* infections by evaluating the in vitro synergistic effects of tigecycline with various antibiotics using the E-test method.

**Materials and methods:**

Synergy testing by E-test was performed with various antibiotic combinations in 10 *S. maltophilia* isolates identified as a cause of infection. The antibiotics used in the study included tigecycline (TGC), cefoperazone-sulbactam (CPS), ceftazidime (TZ), levofloxacin (LEV), and trimethoprim-sulfamethoxazole (cotrimoxazole) (TS). Four different combinations (TGC-CPS, TGC-TZ, TGC-LEV, TGC-TS) were studied with the E-test synergy method.

**Results:**

*S. maltophilia* isolates were found to have the highest level of susceptibility to trimethoprim-sulfamethoxazole, tigecycline, and levofloxacin. The fractional inhibitory concentration (FIC) index was calculated as FIC = MICAB/MICA + MICBA/MICB. The FIC index values were calculated and classified as synergistic (FIC < 0.5), additive (FIC = 0.5–1), indifferent (FIC = 1–4), and antagonistic (FIC > 4). According to FIC index values, synergy was found with the highest rate with TGC-CPS and TGC-LEV combinations (20%). Antagonistic activity was not found in any combination.

**Conclusion:**

When trimethoprim-sulfamethoxazole cannot be used because of resistance or allergy, tigecycline alone or in combination may be included as an alternative option. Although in vitro results are promising, clinical data are required.

## 1. Introduction

*Stenotrophomonas maltophilia* is a nonfermentative bacterium that studies over the last 10 years have shown to be a significant nosocomial pathogen. The most common nosocomial infections caused by* S. maltophilia* are bacteremia and pneumonia, which frequently lead to complications and death (1,2). Studies have shown that the mortality rate of *S. maltophilia* infections increased up to 37.5% in patients who received inappropriate antibiotic treatment as compared to patients who received appropriate antibiotic treatment (3). *S. maltophilia* is intrinsically resistant to β-lactams, quinolones, aminoglycosides, tetracyclines, and disinfectants (4,5). There is no ideal standard treatment. The agent that is used most commonly in treatment is cotrimoxazole (6,7), but it may be contraindicated in cases for causing allergic reaction. There are also reports of cotrimoxazole resistance among *S. maltophilia *isolates that acquired the *sul* gene (8). Levofloxacin is an alternative drug option for treatment of *S. maltophilia* infections (susceptibility rates: 78%–87%) (9,10), but there are reports of fluoroquinolone resistance caused by membrane proteins and efflux pumps (5,7). Because of the potential for resistance development, some authors recommend antibiotic combinations for *S. maltophilia* infection treatment (11).

Tigecycline is the first antibiotic in the glycylcycline group. Tigecycline is approved to treat complicated skin and soft tissue infections, complicated intraabdominal infections, and community-acquired pneumonia. It shows therapeutic activity against gram-positive and gram-negative microorganisms, including those with multidrug resistance (12). In vitro susceptibility rates of tigecycline were found to be high, above 90%, in many studies worldwide (13).

In this study, we aimed to determine the usefulness of tigecycline in combination treatment of *S. maltophilia* infections by evaluating the in vitro effects of combinations of tigecycline with various antibiotics. We used the E-test method for 10 *S. maltophilia* isolates identified as infectious agents.

## 2. Materials and methods

The E-test synergy method was performed using various antibiotic combinations and 10 *S. maltophilia* isolates identified as causative agents. The isolates used in this study were selected from* S. maltophilia* isolates causing nosocomial infection in various services and intensive care units at the Gazi University Hospital between January 2011 and June 2015. The distribution of the clinical specimens of the isolates were as follows: peripheral blood culture 4 (40%), catheter blood culture 2 (20%), ETA (endotracheal aspirate) culture 2 (20%), pleural fluid culture 1 (10%), and bile fluid culture 1 (10%).

### 2.1. In vitro E-test synergy method

The isolates obtained in our study were stored at –80 °C until the study was performed. The isolates were identified using a BBL Crystal Enteric/Nonfermenter ID Kit (Becton Dickinson, USA). 

The minimum inhibitory concentration (MIC) values of tigecycline, cotrimoxazole, levofloxacin, ceftazidime, and cefoperazone-sulbactam were determined using the E-test method. For the E-test, suspensions equivalent to 0.5 McFarland standard were obtained from pure bacterial colonies and inoculated onto Mueller–Hinton medium (Becton Dickinson). E-test strips were prepared for each antibiotic. After incubation for 24 h at 35 °C, MICs were read and interpreted according to the manufacturer’s instructions. To prevent misinterpretation of tigecycline MICs, the agar plates were used within 12 h after preparation. The susceptibility breakpoints for cotrimoxazole, levofloxacin, and ceftazidime were interpreted using the Clinical Laboratory Standard Institute’s criteria for *S. maltophilia*. The cefoperazone-sulbactam susceptibility was determined by the CLSI criteria for Enterobacteriaceae (14). Tigecycline susceptibility was determined using the Food and Drug Administration’s breakpoints for Enterobacteriaceae (15).

For the synergy method, the E-test strip of drug A was applied to the surface of agar plates and left for 1 h at room temperature. Subsequently, the strip was removed and a strip of drug B was applied onto the imprint of strip A. The plates were incubated for 24 h at 35 °C and then the MIC levels of each drug and combination were read. The fractional inhibitor concentration (FIC) index was calculated using the formula FIC = MICAB/MICA + MICBA/MICB. The FIC index was interpreted as follows: synergistic, ≤0.5, additive, >0.5 to <1, indifferent, >1 to ≤4, and antagonistic, >4.

*Pseudomonas aeruginosa* ATCC (American Type Culture Collection) 27853 and *Escherichia coli* ATCC 25922 were used as quality control strains. 

## 3. Results 

The MIC values of the antibiotics among *S. maltophilia* isolates are shown in Table 1. The susceptibilities of the antibiotics were as follows: cotrimoxazole (100%), tigecycline (80%), levofloxacin (80%), ceftazidime (70%), and cefoperazone-sulbactam (50%).

**Table 1:** MIC values of the isolates.

	TGC (D: ≤2 µm/mL)	CPS (D: ≤16 µm/mL)	TS (D: ≤2/38 µm/mL)	TZ (D: ≤8 µm/mL)	LEV (D: ≤2 µm/mL)
1st isolate	0.75	64	0.25	256	0.5
2nd isolate	0.38	64	0.064	256	0.25
3rd isolate	0.5	48	0.047	4	0.5
4th isolate	1	16	0.038	8	0.5
5th isolate	4	6	0.047	1	4
6th isolate	0.38	16	0.125	8	0.5
7th isolate	4	96	0.064	256	8
8th isolate	0.5	4	0.032	3	0.5
9th isolate	0.25	48	0.032	3	0.25
10th isolate	2	3	0.125	0.75	0.25

TGC: Tigecycline, TS: Trimethoprim-sulfamethoxazole, LEV: levofloxacin CPS: cefoperazone-sulbactam, TZ: ceftazidime.

In vitro interactions (synergic, additive, indifferent, and antagonistic) of the 4 combinations studied (TGC-CPS, TGC-TZ, TGC-LEV, TGC-TS) according to FIC results are shown in Table 2. Synergy was found with the highest rate in TGC-CPS and TGC-LEV combinations (20%) and antagonistic activity was not found in any combination.

**Table 2:** The results of in vitro interactions of 4 antibiotic combinations.

Antibiotic combinations	Synergic number (%)	Additive number (%)	Indifferent number (%)	Antagonist number (%)
TGC-CPSTGC-TZTGC-LEVTGC-TS	2 (20)1 (10)2 (20)0 (0)	4 (40)4 (40)1 (10)2 (20)	4 (40)5 (50)7 (70)8 (80)	0 (0)0 (0)0 (0)0 (0)

TGC: Tigecycline, TS: trimethoprim-sulfamethoxazole, LEV: levofloxacin, CPS: cefoperazone-sulbactam, TZ: ceftazidime.

## 4. Discussion 

*S. maltophilia* has a high level of intrinsic resistance to β-lactams, quinolones, aminoglycosides, tetracyclines, disinfectants, and heavy metals. Management of these infections is difficult because of resistance to many antimicrobial agents. The therapeutic agent recommended for *S. maltophilia* is cotrimoxazole. Some recent studies have instead recommended antimicrobial combination therapies, especially for patients with septic shock or neutropenia, immunocompromised patients, and patients intolerant of cotrimoxazole, but only a few studies have been focused on these antibiotic combinations (16).

Zelenitsky et al. conducted a study comparing cotrimoxazole monotherapy and its combinations with various antibiotics (ciprofloxacin, ceftazidime, gentamycin, and tobramycin) in 4 clinical isolates in an in vitro pharmacodynamic infection model. They found that cotrimoxazole worked as a bacteriostatic agent against all isolates when given alone, and all combinations of cotrimoxazole were more active than monotherapy as determined by bacterial reductions at both 24 and 48 h. They concluded that their preclinical data supported further investigation of antibiotic combinations in the treatment of serious *S. maltophilia* infections (17).

In clinical *S. maltophilia* isolates, tetracycline derivatives minocycline, doxycycline, and tigecycline have been shown to have high in vitro efficacy. There is a very little evidence of their use in treatment, however (6). Tigecycline, a wide-spectrum glycylcycline derivative, may overcome tetracycline resistance related to efflux pumps and ribosomal target modification. Studies have found that tigecycline is effective for strains resistant to cotrimoxazole (18,19). In a global study evaluating 1586 isolates, the susceptibility rates were 96% for cotrimoxazole and 95.5% for tigecycline (13). In a study conducted by Church et al., 17% of the *S. maltophilia* isolates were resistant to cotrimoxazole. Minocycline, tigecycline, and colistin had the highest efficacy. Colistin and tigecycline combination produced the best results (20). In another study conducted by Wei et al., synergism and antagonism were not detected in tigecycline + cotrimoxazole and tigecycline + ceftazidime combinations. All of the isolates showed indifferent activity. While synergy was found in a few isolates in the tigecycline and moxifloxacin combination, antagonistic action was not detected in any combination (21).

According to the results of in vitro studies, tigecycline could be considered an alternative option in the treatment of *S. maltophilia* infections, especially in combination therapy (22). However, the choice between monotherapy and combination therapy remains controversial. In a study performed by Tekce et al., the efficacy of tigecycline treatment was compared with cotrimoxazole in nosocomial *S. maltophilia* infections over a 3-year period. Clinical improvement was similar in the two groups: 69.2% in the cotrimoxazole group and 68.4% in the tigecycline group. The authors concluded that tigecycline can be considered as an alternative option in the treatment of *S. maltophilia* infections (23). Apart from this study, anecdotal evidence about the use of tigecycline in treatment has been reported in some studies. There are no data about the use of this agent in combination in clinical practice (24–26).

In our study, cotrimoxazole showed the lowest MIC levels against *S. maltophilia* isolates, followed by levofloxacin and tigecycline. We also evaluated tigecycline in combination with 4 different antibiotics. The best results were obtained with TGC + CPS and TGC + LEV combinations. Additive interaction was detected mainly in the TGC + CPS combination. In vitro synergy studies do not show the effects of antibiotic pharmacodynamics and host immune response, but synergistic combinations (TGC + CPS and TGC + LEV) may still be a therapeutic option in certain *S. maltophilia* infections. Our results should be supported with clinical studies.

## References

[ref0] (2004). Stenotrophomonas maltophilia: the significance and role as a nosocomial pathogen. J Hosp Infect.

[ref1] (2015). Hsueh PR. Update on infections caused by Stenotrophomonas maltophilia with particular attention to resistance mechanisms and therapeutic options. Front Microbiol.

[ref2] (2009). Attributable mortality of Stenotrophomonas maltophilia infections: a systematic review of the literature. Future Microbiol.

[ref3] (1997). Multiple antibiotic resistance in Stenotrophomonas maltophilia. Antimicrob Agents Chemother.

[ref4] (2000). Multiple antibiotic resistance in Stenotrophomonas maltophilia: involvement of a multidrug efflux system. Antimicrob Agents Chemother.

[ref5] (2009). Stenotrophomonas maltophilia: an emerging opportunist human pathogen. Lancet Infect Dis.

[ref6] (2007). Update on Stenotrophomonas maltophilia infection in the ICU. Clin Pulm Med.

[ref7] (2007). Global emergence of trimethoprim/sulfamethoxazole resistance in Stenotrophomonas maltophilia mediated by acquisition of sul genes. Emerg Infect Dis.

[ref8] (2000). Levofloxacin in vitro activity and time-kill evaluation of Stenotrophomonas maltophilia clinical isolates. J Antimicrob Chemother.

[ref9] (2008). Antimicrobial susceptibility profile of contemporary clinical strains of Stenotrophomonas maltophilia isolates: can moxifloxacin activity be predicted by levofloxacin MIC results?. J Chemother.

[ref10] (2008). Therapeutic options for Stenotrophomonas maltophilia infections beyond co-trimoxazole: a systematic review. J Antimicrob Chemother.

[ref11] (2009). Safety and efficacy of intravenous tigecycline in treatment of community-acquired pneumonia: results from a double-blind randomized phase 3 comparison study with levofloxacin. Diagn Microbiol Infect Dis.

[ref12] (2010). Antimicrobial susceptibilities of a worldwide collection of Stenotrophomonas maltophilia isolates tested against tigecycline and agents commonly used for S. maltophilia infections. Antimicrob Agents Chemother.

[ref13] (2012). Performance Standards for Antimicrobial Susceptibility Testing; Twenty-Second Informational Supplement. CLSI Document M100-S23.

[ref14] (2005). Tygacil (Tigecycline) for Injection [Package Insert].

[ref15] (2013). Antimicrobial susceptibility of Stenotrophomonas maltophilia isolates from Korea, and the activity of antimicrobial combinations against the isolates. J Korean Med Sci.

[ref16] (2005). Antibiotic combinations significantly more active than monotherapy in an in vitro infection model of Stenotrophomonas maltophilia. Diagn Microbiol Infect Dis.

[ref17] (2005). Antimicrobial activity of tigecycline tested against nosocomial bacterial pathogens from patients hospitalized in the intensive care unit. Diagn Microbiol Infect Dis.

[ref18] (2007). In vitro activity of tigecycline against clinical isolates of Acinetobacter baumannii and Stenotrophomonas maltophilia. J Antimicrob Chemother.

[ref19] (2013). Antimicrobial susceptibility and combination testing of invasive Stenotrophomonas maltophilia isolates. Scand J Infect Dis.

[ref20] (2016). Evaluation of trimethoprim/sulfamethoxazole (SXT), minocycline, tigecycline, moxifloxacin, and ceftazidime alone and in combinations for SXT-susceptible and SXT-resistant Stenotrophomonas maltophilia by in vitro time-kill experiments. PLoS One.

[ref21] (2007). Antimicrobial therapy for Stenotrophomonas maltophilia infections. Eur J Clin Microbiol Infect.

[ref22] (2012). Tigecycline as a therapeutic option in Stenotrophomonas maltophilia infections. J Chemother.

[ref23] (2009). Successful treatment of Stenotrophomonas maltophilia soft tissue infection with tigecycline: a case report. J Chemother.

[ref24] (2008). Tigecycline for treatment of nosocomial acquired pneumonia possibly caused by multi-drug resistant strains of Stenotrophomonas maltophilia. J Chemother.

